# Home environment and frailty in very old adults

**DOI:** 10.1007/s00391-021-01969-6

**Published:** 2021-09-27

**Authors:** Jaroslava Zimmermann, Sylvia Hansen, Michael Wagner

**Affiliations:** 1 ceres – Cologne Center for Ethics, Rights, Economics, and Social Sciences of Health, University of Cologne, Cologne, Germany; 2 Institute of Sociology and Social Psychology, University of Cologne, Cologne, Germany

**Keywords:** Frail, Environmental factors, Community dwelling, Nursing home, Germany, Gebrechlich, Umweltbezogene Faktoren, Gemeinschaftliches Wohnen, Pflegeeinrichtung, Deutschland

## Abstract

**Background:**

Since older adults spend much time in their home environment (HE), frailty may occur as a consequence of a maladaptation to the HE. The aim of this study was to describe the prevalence of frailty in the very old population of North Rhine-Westphalia, and to examine the association between the HE and the frailty levels of these individuals.

**Methods:**

Data from a cross-sectional representative study were used, including data on 1577 community-dwelling individuals and nursing home residents aged ≥ 80 years. Objective and subjective HE aspects were included. Frailty was defined according to four criteria: exhaustion, unintentional weight loss, weakness, and low physical activity. Adjusted multinomial regression modelling was used to analyze the link between the HE and frailty levels.

**Results:**

Of the very old individuals, 24.3% were robust, 57.0% were prefrail, and 18.7% were frail. Adjusting for relevant sociodemographic and health characteristics, being not closely attached to the HE was linked with an increased probability of being prefrail and frail. An improvement of the residential area was associated with a decrease in odds of being frail. Living in communities with less than 50,000 and with 100,000–499,999 inhabitants decreased the odds of being frail.

**Discussion:**

Frailty prevalence is shown to be higher in the very old population than in the younger age groups in Germany. Early identification of frailty and tailored interventions focused on improving objective and subjective attributes of the HE are needed to reduce the risk of frailty.

**Supplementary Information:**

The online version of this article (10.1007/s00391-021-01969-6) contains supplementary material, which is available to authorized users.

## Abstract (de)

**Hintergrund:**

Da ältere Menschen viel Zeit in ihrer Wohnumgebung (HE) verbringen, kann als Folge einer Fehlanpassung an HE Gebrechlichkeit auftreten. Das Ziel der Studie war, die Gebrechlichkeitsprävalenz in der Hochaltrigenpopulation Nordrhein-Westfalens zu beschreiben und den Zusammenhang zwischen dem HE und Gebrechlichkeitsniveau zu untersuchen.

**Methoden:**

Es wurden Daten aus einer repräsentativen Querschnittsstudie verwendet. Insgesamt wurden Daten von 1577 in Privathaushalten und Heimen lebenden Personen im Alter von ≥ 80 Jahren analysiert. Einbezogen wurden objektive und subjektive Aspekte von HE. Gebrechlichkeit wurde anhand von vier Kriterien definiert: Erschöpfung, unabsichtlicher Gewichtsverlust, Schwäche, niedrige körperliche Aktivität. Mit einem adjustierten multinomialen Regressionsmodell wurde der Zusammenhang zwischen HE und Gebrechlichkeitsniveau geschätzt.

**Ergebnisse:**

In der Hochaltrigenpopulation waren 24,3 % robust, 57,0 % prägebrechlich und 18,7 % gebrechlich. Unter Berücksichtigung relevanter soziodemographischer und gesundheitlicher Merkmale war die fehlende Verbundenheit mit HE mit einer erhöhten Wahrscheinlichkeit assoziiert, prägebrechlich und gebrechlich zu sein. Eine Verbesserung der Wohngegend war mit einer verminderten Wahrscheinlichkeit des Auftretens von Gebrechlichkeit verbunden. Der Wohnsitz in Gemeinden mit weniger als 50.000 und 100.000 bis 499.999 Einwohnern reduzierte die Wahrscheinlichkeit, gebrechlich zu sein.

**Diskussion:**

Die Gebrechlichkeitsprävalenz war in der Hochaltrigenpopulation höher als in den jüngeren Altersgruppen in Deutschland. Frühzeitige Identifikation und gezielte Interventionen, die sich mit Verbesserungen objektiver und subjektiver Merkmale von HE befassen, sind erforderlich, um das Gebrechlichkeitsrisiko zu senken.

## Introduction

Environmental gerontology proposes that people’s home environment (HE) influences their quality of life at older ages, since older people spend most of their time at home [28]. If an individual’s loss of competence can no longer be compensated for by the existing HE conditions, or by the person’s subjective HE experience because of limited coping options, a so-called person-environment misfit occurs [28]. This imbalance can lead to increased stress, restricted autonomy and health decline [28]. As frailty is characterized by an increased vulnerability to external stressors [13] and is associated with adverse health outcomes, including mortality [2, 11], hospitalization [16, 19] and nursing home admission [6, 16], we assume that unfavorable objective HE conditions and negative HE experiences are associated with frailty status in very old adults (VOA). In line with the “Challenges and Potentials Model of Quality of Life in Very Old Age” [27], this study focused on the link between environmental conditions and individual resources.

Two systematic reviews [9, 12] have confirmed that living in socioeconomically deprived communities is associated with frailty; most of the studies used Fried’s definition of frailty [11], known as the frailty phenotype (FP). Caldwell et al. [5] reported that the physical deterioration of buildings in close proximity to peoples’ places of residence can have a negative impact on FP, whereas feeling secure and connected to one’s neighborhood is associated with lower FP levels [5, 12]. A longitudinal study [30] found that having a higher proportion of green spaces in the HE can be linked directly with improvements in FP, as well as indirectly through increased physical activity. In addition, higher levels of subjective esthetic quality and walkability of the HE was reported to have a protective effect against frailty [29]. A few studies found that neglected interior living space can have a negative impact on an individual’s health status, physical and cognitive functioning, suggesting a poorly maintained HE as an indicator of a person’s frailty status [8, 10].

There is broad evidence that frailty increases with age [7, 21]. In Germany, a representative study of community-dwelling older adults reported a FP prevalence of 2.6% in the population aged 65–79 years [4], while another study found a FP prevalence of 12.1% in those over age 65 years [24]. The differences in these findings are attributable in part to their methodologies [4]. As people who are aged 80 years or older or are institutionalized tend to be underrepresented in or even excluded from frailty research [7], the first aim of this study was to analyze FP prevalence in the very old population of North Rhine-Westphalia (NRW). Based on the given research evidence, the second aim was to examine the association between subjective and objective aspects of the HE and the FP levels in VOA.

## Methods

### Study design

Data from the representative study “Quality of Life and Well-Being of the Very Old in North Rhine-Westphalia” (NRW80+) of individuals aged 80 years or older were analyzed. The study sample included community-dwelling individuals as well as nursing home residents. If the target person could not be interviewed due to health impairments, proxy interviews were conducted. Data weightings were applied to correct for oversampling in older age groups (85–89 years, 90 years or older) and men. The results are applicable to the very old population in NRW. In total, 1863 computer-assisted personal interviews were conducted, including 176 interviews with proxies. Further information about the study design has been published elsewhere [14].

### Dependent variable

#### Frailty.

In line with most previous studies [9, 12], the frailty definition used in this study is based on Fried’s FP [11], taking into account four instead of five established Fried frailty criteria [2, 19]: exhaustion, unintentional weight loss, weakness, and low physical activity. Individuals who met three or four criteria were classified as frail; those who met one or two criteria were categorized as prefrail; while those who met none of the four criteria were considered robust. Exhaustion was assessed by two items: “How strongly do you notice having less energy with increasing age?” and “How strongly do you notice having to restrict your activities with your increasing age?”. Participants who responded with “strong” or “very strong” to at least one of the items were classified as exhausted. Participants who answered “yes” to the question “Have you unintentionally lost a considerable amount of weight in the last 12 months?” met the criterion for unintentional weight loss. Weakness was measured with a hand-held dynamometer (Smedley, TTM, Tokyo, Japan, 100 kg). Participants whose highest value of grip strength was within the lowest quantile of the overall sample (women < 15 kg; men < 26 kg) were considered weak. Additionally, individuals who were identified by the interviewers as “not being able to perform grip strength test” were classified as weak. Physical activity was assessed using a combination of physical activity variables. Participants answered questions about whether and how often they carried out the following activities: sports (e.g., swimming), going for a walk, being physically active in general, and having hobbies such as gardening. Hobbies were categorized as involving either no/low physical activity (reading, handcrafts) or moderate/vigorous physical activity (gardening, skiing) using metabolic equivalent of tasks (MET) minutes, as recommended by the Ainsworth compendium [1]. The VOA were assigned to the low physical activity group if they did not participate in any of these activities, only engaged in hobbies with no or low physical activity or participated in activities only two times per week or less^1^. The VOA who engaged in any (combination of) activities^1^ three or more times per week were categorized as being at least moderately active. This cut-off leads to 41.8% of VOA with no/low physical activity, which is comparable to those reported in other studies [25].

### Independent variables

#### Home environment.

Interviewers evaluated the participants’ interior living space condition, the attractiveness of their residential area, and their place of residence. Two subjective evaluations of the HE were included. In the case of walkability, participants were asked: “How suitable is your external living environment for walking, using a wheelchair, or managing things?”. The participants’ perceived attachment to outdoor place was assessed using one item: “How closely do you feel connected to your living environment?”. Furthermore, the community type was considered using the BIK classification of seven region types [3]. Since the proportion of communities classified as less than 2000 and 2000–4999 inhabitants is 3% in NRW [3], these were not represented in the study sample. The categories 5000–19,999 and 20,000–49,999 inhabitants were merged. Control variables are listed in Table 1; more details are provided in Supplement 1.

**Table 1:** Descriptive sample characteristics according to frailty level (*N* = 1588)

Variable	Total	Robust	Prefrail	Frail
	*% or mean (SD)*	*% or mean (SD)*	*% or mean (SD)*	*% or mean (SD)*
*Total*	*100*	*24.3*	*57.0*	*18.7*
Age (years)***	84.9 (4.0)	83.5 (3.3)	85.1 (4.0)	85.9 (4.4)
Sex**				
Male	36.8	42.0	36.8	30.2
Female	63.2	58.0	63.2	69.8
*Socioeconomic status* **	41.4 (20.6)	43.6 (21.1)	41.4 (20.3)	38.5 (20.9)
Relationship status***				
No partnership	58.5	47.1	60.2	68.1
In partnership	41.5	52.9	39.8	31.9
*Migration background*				
Yes	23.4	20.2	24.8	23.1
No	76.6	79.8	75.2	76.9
Self-rated health status***				
Very good	10.9	22.7	8.9	1.7
Rather good	50.4	60.5	52.0	32.4
Rather poor	31.4	15.3	32.4	49.4
Very poor	7.3	1.6	6.8	16.5
Number of chronic diseases***	3.4 (2.3)	2.3 (1.9)	3.5 (2.3)	4.5 (2.3)
Instrumental activities of daily living***	1.5 (0.6)	1.9 (0.3)	1.5 (0.5)	1.0 (0.6)
Walkability*				
Rather high/high	66.0	69.6	66.5	59.4
Rather low/low	34.0	30.4	33.5	40.6
Residential area***				
Good/very good	65.7	72.8	65.5	57.2
Moderate	31.7	26.1	31.9	38.4
Rather poor/very poor	2.6	1.0	2.7	4.4
Condition of interior living space***				
Well kept	62.6	71.4	63.1	49.7
Moderately kept	35.1	27.3	34.4	47.4
Neglected	2.3	1.3	2.5	2.9
Attachment to outdoor place***				
Rather close/very close	77.2	84.7	77.1	67.5
Not very close/not close at all	22.8	15.3	22.9	32.5
Place of residence***				
Private household	89.3	94.0	89.9	81.2
Nursing home	10.7	6.0	10.1	18.8
Community type***				
5000–49,999	13.6	14.1	14.9	9.4
50,000–99,999	13.3	9.8	13.7	16.4
100,000–499,999	34.3	37.8	35.4	26.7
≥ 500,000	38.8	38.3	36.1	47.5

Weighted data. In case the values differed among the imputed datasets, average values are reported. The χ^2^-test was applied to ordinal and categorical variables. Kruskal-Wallis test (adjusted for multiple comparison) to was applied to continuous variables. Details on control variables are given in Supplement 1

*SD* standard deviation

*p < 0.05, **p < 0.01, ***p < 0.001

### Statistical analyses

In the current study, 286 participants were excluded: handgrip strength was not measured in interviews with proxies (*N* = 176), and 110 respondents refused to perform the test. The final subsample included 1577 persons. Multiple imputation was used to replace missing values in all dependent and independent variables. In total, there were 128 missing values. Further information on the multiple imputation are presented in Supplement 1. Unless otherwise specified, the reported results are based on the fully imputed dataset. A multinomial regression model was estimated to analyze the relationship between the HE aspects and the FP level adjusted for health status and sociodemographics. All analyses were performed with IBM SPSS Statistics version 26 (SPSS Inc., Chicago, IL, USA). The variance inflation factors did not exceed the threshold of two by any of the variables. Statistical significance was determined as *p* < 0.05.

## Results

Of the VOA in NRW, 24.3% (95% confidence interval, CI, 22.3–26.5%) were classified as robust, 57.0% (95% CI 54.6–59.4%) were found to be prefrail, and 18.7% (95% CI 16.9–20.7%) were found to be frail. Sample descriptions according to the four frailty criteria are provided in Supplement 2. As shown in Table 1, the frailty groups differed significantly in most characteristics. Frail persons were predominantly older, women, had lower socioeconomic status, no partner, poorer health status, more chronic diseases, needed more help with instrumental activities of daily living, rated walkability of external living environment less frequently as rather high/high, lived more frequently in poor residential areas, in neglected interior living space, were not very closely/not closely at all attached to their outdoor place, lived in nursing homes, and in communities with 500,000 or more inhabitants compared to robust and prefrail very old adults.

In the fully adjusted model (Table 2), holding all other variables constant, an improvement in the residential area (e.g., from rather poor/very poor to moderate) was statistically significant associated with a 41.6% decrease in odds of being frail. Being not very closely/not closely at all (compared to rather close/very close) attached to the outdoor place increased the odds of being prefrail by 62.7% and being frail by 131.7%. Living in communities with less than 50,000 (compared to 500,000 or more) inhabitants decreased the odds of being frail by 71.2%. Similarly, actual residence in communities with 100,000–499,999 inhabitants was linked to a 49.7% decrease in odds of being frail. The model estimates are presented in Table 2. A sensitivity analysis performed with community-dwelling individuals only revealed comparable results. For more details see Supplement 2.

**Table 2:** Multinomial regression model of the associations between home environment and frailty level (*N* = 1575^a^)

Variable	Prefrail relative to robust			Frail relative to robust		
	*Odds ratio*	*95% CI*		*Odds ratio*	*95% CI*	
*Walkability (Ref: rather high/high)*	1.010	0.754	1.352	0.790	0.524	1.191
*Residential area*	0.807	0.607	1.074	0.584**	0.396	0.860
*Condition of interior living space*	0.884	0.664	1.178	0.802	0.542	1.187
*Attachment to outdoor place (Ref: rather close/very close)*	1.627**	1.144	2.316	2.317***	1.452	3.696
*Place of residence (Ref: private household)*	0.623	0.340	1.143	0.488	0.231	1.032
*Community type (Ref: ≥* *500,000)*						
5000–49,999	0.944	0.613	1.454	0.288***	0.149	0.555
50,000–99,999^b^	1.471	0.926	2.337	0.966	0.517	1.803
100,000–499,999	1.077	0.789	1.470	0.503**	0.315	0.802
*Pseudo R*^2^	0.420***					

Weighted data. The model has been additionally adjusted for age, sex, socioeconomic status, relationship status, migration background, self-rated health status, number of chronic diseases and instrumental activities of daily living. See Supplement 2 for further details on the model estimates

*Ref* reference category, *CI* confidence interval

*p < 0.05, **p < 0.01, ***p < 0.001

^a^Final analysis sample with observed values for dependent variable

^b^The results differed between the original and full imputed datasets. In the original data, living in communities with 50,000–99,999 inhabitants was significantly associated with increased odds of being pre-frail (odds ratio, OR = 1.776, 95% CI 1.086–2.903)

^c^Calculated as the mean value of the pseudo R^2^ from all imputed datasets

## Discussion

Our analysis showed that at 18.7%, the FP prevalence of the very old population of NRW was higher than that of younger older adults in Germany [4, 24]. On the one hand, this confirmed the widespread empirical evidence that the risk of prefrailty and frailty increases with age [7, 21]. On the other hand, the inclusion of nursing home residents could contribute to the higher FP prevalence (cf. [6]). In other studies, nursing home residents were excluded [4, 6, 16]. Furthermore, our findings support the assumptions of environmental gerontology [28]: after adjusting for relevant individual health and socioeconomic characteristics, the quality of the respondents’ residential location (as assessed by interviewers), as well as their subjective attachment to their HE, remained significant predictors of their FP levels. Consistent with previous studies [5, 12], not feeling connected to one’s own HE was associated with a higher likelihood of prefrailty and frailty. Wahl and Oswald [28] argued that particularly at older ages, people’s subjective perception of their HE tends to reflect their personal identities. The failure to establish an attachment to the HE over a longer period of time may have a negative impact on an individual’s health and psychological well-being [28], which may, in turn, lead to the development of frailty.

In line with previous research [9, 12], our results showed that VOA who lived in poorer residential areas were more likely to be classified as frail. Thus, following Wahl and Oswald [28], frailty might be seen as an outcome of person-environment misfit. For example, VOA who are functionally impaired may be unable to overcome an unfavorable HE (such as deteriorated footpaths), which can lead to reduced mobility outside the home or facility, and, in turn, to an increased risk of frailty [5, 30]. Although there is an empirical evidence that an individual’s neighorhood affects frailty risk, conceptual frameworks explicitly explaining this relationship are still missing [9, 12]. Unlike in previous studies [20], our study did not confirm the association between individual socioeconomic status and frailty, supporting the age as leveller theory suggesting that socioeconomic disparities in health diminish in very old age [17].

Inconsistent with previous studies reporting a higher likelihood of being frail among older population in rural areas [18, 22], we found that VOA who were frail more likely lived in metropolitan communities. NRW is characterized by high urbanization levels [3], therefore, rural communities were not included in our study sample. Since there are no existing studies of regional differences in frailty levels in Germany, this association should be further investigated using national samples.

In this study, a few aspects of the HE (walkability, place of residence, condition of the interior living space) were not found to be associated with FP. From a person-environment-fit perspective [28], older individuals who are able to cope with HE challenges can protect themselves from the negative effects of these shortcomings on their health by adapting their behavior and routines. Hladek et al. [15] found that a high level of coping self-efficacy can protect older people against prefrailty, independent of the individual health deficits. Thus, our results might indicate that VOA in NRW had sufficient coping capacities to enable them to adjust to these HE barriers. In further research, the moderation effects of coping efficacy on the link between HE and FP should be explored.

The present study has provided initial representative results on the FP prevalence and its relationship to the HE in VOA in Germany. Subjective as well as objective evaluations were used to precisely operationalize the HE. The definition of frailty we used was based on Fried et al. [11] to ensure that our findings were comparable to those of other studies; however, minor modifications were necessary due to data availability. First, because the walking speed was not assessed in the NRW80+, four FP criteria were considered. Previous research has shown that four symptoms can predict the adverse health outcomes associated with frailty [2, 19], and the exclusion of the criterion of walking speed has the smallest impact on predicting mortality [26]. Second, we used frequency measures to operationalize physical activity, which differed from the original questionnaire based on expended kilocalories per week [11]. The original questionnaire was recognized as being less suitable for vulnerable old individuals, as it was developed primarily for the younger population, and has been validated only for men and healthy older adults [26]. Furthermore, interviews with proxies had to be excluded from the analyses because it was not possible to measure the handgrip strength of the target person. While the FP prevalence might be underestimated in this study because the proxy interviews were conducted in cases in which the target persons were suffering from health impairments, it was still found to be higher than in other German studies [4, 24]. To minimize the possible bias, multiple imputation was applied to replace the missing values.

## Practical recommendations

- Based on our data, 523,331 prefrail and 171,705 frail individuals can be expected in very old population of NRW (collection period 2017/2018).
- The findings revealed that certain HE characteristics are associated with the FP risk. In contrast to individual risk factors for frailty, such as poor health or older age, the HE might be modifiable.
- Frailty risk can be reduced through early identification and appropriate interventions involving the promotion of physical activity [23]. Therefore, efforts should be made at the community and federal state levels to enable and promote the mobility of vulnerable older adults outside their homes or facilities, e.g., through the adaptation of public transport to ensure accessibility, the adequate extension and maintenance of footpaths, street lighting improvements, or support for group activities in the immediate HE.

## Supplementary Information

Supplementary Information on methodological issue

Supplementary information on results

## Footnotes

1 Hobbies were counted as activity if categorized as moderate/vigorous physical activity.
