# Supplementary material for: Home environment and frailty in very old adults
Source: Z Gerontol Geriatr. 2021 Sep 27;54(Suppl 2):114–9. doi: 10.1007/s00391-021-01969-6 (PMC8551134; doi:10.1007/s00391-021-01969-6)
Supplement: Supplementary file 1 — Supplementary Information on methodological issue [file 391_2021_1969_MOESM1_ESM.docx]

**Supplementary Information on methodological issue**

*Description of included control variables*

Based on SF-8 [1], *self-rated health status* was assessed with one item rated as very good, rather good, rather poor, and very poor. *Number of chronic diseases* was calculated based on self-reported medically treated diseases. The score ranged from zero to 19. Functional abilities were measured with seven items referring to *instrumental activities of daily living* [4]. The mean score was calculated with a range from zero (not possible without help) to two (no help needed). Additionally, the following sociodemographic characteristics were considered: *age* (in years), *gender*, *relationship status* (in partnership vs. no partnership), *migration background*, and *socioeconomic status* *(SES).* SES referred to the participant’s last profession before retirement based on the lnternational Standard Classification of Occupations 2008 [2]. The SES scale ranges from 16 (e.g. helpers, cleaners, agricultural laborers) to 90 (judges), the detailed information on the construction and interpretation of the scale are provided in the publication of Ganzeboom and Treiman [3]. In Table 1, the mean value for socioeconomic status was 41.4. Following the classification of Ganzeboom and Treiman [3], the scale value of 41 refers for example to electronics mechanics or locomotive-engine drivers, the value 42 refers for example to aircraft engine mechanics & fitters or firefighters. Detailed information about the survey intruments used in NRW80+ are available elsewhere [5].

*Further details about multiple imputation approach*

In the analysis sample, there were 8 missing values in the measure of exhaustion, 8 in unintentional weight loss, 3 in the measure of weakness, and 11 in low physical activity. Table S1 provides detailed information on the number of missing values. Using logistic regression models, predictors of missing values for all frailty indicators were examined. In this way, in addition to variables described in Table 1, four other predictors of frailty (body mass index, basic activities of daily living, use of wheelchairs as well as overal life satisfaction) were identified and consequently included in the imputation model. Basic activities of daily living were assessed using seven items (cf.[4]). An mean score was calculated, ranging from zero (not possible without help) to two (no help needed). The use of wheelchairs was collected in the form of a dichotomous variable (yes/no). The overall life satisfaction was measured on a scale ranging from zero (completely dissatisfied) to 10 (completely satisfied). Twenty imputed datasets were generated. The results obtained from the original (non-imputed) dataset are only reported if they significantly differed from the imputed dataset.

Table S1. Description of missing values in the analysis sample

| **Variable** | **Number of missing values** |
| --- | --- |
| Exhaustion | 8 |
| Unintentional weight loss | 8 |
| Weakness | 3 |
| Low physical activity | 11 |
| Age | 0 |
| Sex | 0 |
| Socioeconomic status | 45 |
| Relationship status | 1 |
| Migration background | 0 |
| Self-rated health status | 4 |
| Number of chronic diseases | 17 |
| Instrumental activities of daily living | 0 |
| Walkability | 3 |
| Residential area | 0 |
| Condition of interior living space | 20 |
| Attachment to outdoor place | 8 |
| Place of residence | 0 |
| Community type | 0 |
| Total | 128 |

**References**

1. Ellert U, Lampert T, Ravens-Sieberer U (2005) Messung der gesundheitsbezogenen Lebensqualität mit dem SF-8. Eine Normstichprobe für Deutschland. Bundesgesundheitsblatt, Gesundheitsforschung, Gesundheitsschutz 48:1330–1337. https://doi.org/10.1007/s00103-005-1168-5.

2. Ganzeboom HBG (2010) A new international socio-economic index [ISEI] of occupational status for the international standard classification of occupation 2008 [ISCO-08] constructed with data from ISSP 2002-2007. With an analysis of quality of occupational measurement in ISSP, Lisbon.

3. Ganzeboom HBG, Treiman DJ (1996) Internationally Comparable Measures of Occupational Status for the 1988 International Standard Classification of Occupations. Social Science Research 25:201–239.

4. Lawton MP, Brody EM (1969) Assessment of older people: self-maintaining and instrumental acitities of daily living. Gerontologist 9:179–186.

5. Zank S, Woopen C, Wagner M et al. (2020) Quality of Life and Well-being of Very Old People in NRW (Representative Survey NRW80+). GESIS Data Archive, Cologne. ZA7558 Data file Version 1.0.0, https://doi.org/10.4232/1.13527.
