# Supplementary material for: Home environment and frailty in very old adults
Source: Z Gerontol Geriatr. 2021 Sep 27;54(Suppl 2):114–9. doi: 10.1007/s00391-021-01969-6 (PMC8551134; doi:10.1007/s00391-021-01969-6)
Supplement: Supplementary file 2 — Supplementary information on results [file 391_2021_1969_MOESM2_ESM.docx]

**Supplementary information on results**

Table S2. Descriptive characteristics of the four frailty criteria

| **Frailty criterion** | **Total** | **Women** | **Men** | **Private household** | **Nursing home** |
| --- | --- | --- | --- | --- | --- |
|  | *(N=1,588)%* | *(N=1,003)%* | *(N=585)%* | *(N=1,419)%* | *(N=169)%* |
| Total | 100 | 63.2 | 36.8 | 89.3 | 10.7 |
| Exhaustion | 58.9 | 60.9 | 55.5 | 56.9 | 75.8 |
| Unintentional weight loss | 12.9 | 13.6 | 11.8 | 12.8 | 14.4 |
| Weakness | 30.0 | 32.3 | 26.0 | 27.5 | 50.4 |
| Low physical activity | 41.8 | 44.1 | 37.7 | 40.2 | 54.9 |

|  |
| --- |

| **Variable** | | **Pre-frail relative to robust** | | | | | **Frail relative to robust** | | | | |  |
| --- | --- | --- | --- | --- | --- | --- | --- | --- | --- | --- | --- | --- |
|  |  | *Regression coefficient* | *Standard error* | *Odds ratio* | *95% CI* | | *Regression coefficient* | *Standard error* | *Odds ratio* | *95% CI* | |  |
| Age | | 0.072 | 0.021 | 1.074*** | 1.031 | 1.119 | 0.064 | 0.028 | 1.066* | 1.010 | 1.126 |  |
| Sex (Ref: female) | | 0.088 | 0.162 | 1.092 | 0.796 | 1.500 | 0.103 | 0.236 | 1.108 | 0.698 | 1.759 |  |
| Socioeconomic Status | | 0.003 | 0.004 | 1.003 | 0.996 | 1.010 | 0.006 | 0.005 | 1.006 | 0.996 | 1.017 |  |
| Relationship status (Ref: in partnership) | | 0.283 | 0.160 | 1.327 | 0.970 | 1.816 | 0.319 | 0.235 | 1.376 | 0.868 | 2.183 |  |
| Migration background (Ref: no)^b^ | | 0.229 | 0.166 | 1.257 | 0.908 | 1.740 | -0.030 | 0.238 | 0.971 | 0.609 | 1.547 |  |
| Self-rated health status | | -0.592 | 0.110 | 0.553*** | 0.446 | 0.686 | -1.175 | 0.149 | 0.309*** | 0.231 | 0.413 |  |
| Number of chronic diseases | | 0.166 | 0.039 | 1.180*** | 1.093 | 1.274 | 0.240 | 0.050 | 1.271*** | 1.152 | 1.402 |  |
| Instrumental activities of daily living | | -1.759 | 0.257 | 0.172*** | 0.104 | 0.285 | -3.430 | 0.287 | 0.032*** | 0.018 | 0.057 |  |
| Walkability (Ref: rather high/high) | | 0.010 | 0.149 | 1.010 | 0.754 | 1.352 | -0.236 | 0.209 | 0.790 | 0.524 | 1.191 |  |
| Residential area | | -0.214 | 0.145 | 0.807 | 0.607 | 1.074 | -0.538 | 0.198 | 0.584** | 0.396 | 0.860 |  |
| Condition of interior living space | | -0.123 | 0.146 | 0.884 | 0.664 | 1.178 | -0.220 | 0.200 | 0.802 | 0.542 | 1.187 |  |
| Attachment to outdoor place  (Ref: rather close/very close) | | 0.487 | 0.180 | 1.627** | 1.144 | 2.316 | 0.840 | 0.238 | 2.317*** | 1.452 | 3.696 |  |
| Place of residence (Ref: private household) | | -0.473 | 0.309 | 0.623 | 0.340 | 1.143 | -0.717 | 0.382 | 0.488 | 0.231 | 1.032 |  |
| Community type (Ref: ≥500,000) | |  |  |  |  |  |  |  |  |  |  |  |
|  | 5,000 to 49,999 | -0.058 | 0.220 | 0.944 | 0.613 | 1.454 | -1.246 | 0.336 | 0.288*** | 0.149 | 0.555 |  |
|  | 50,000 to 99,999^c^ | 0.386 | 0.236 | 1.471 | 0.926 | 2.337 | -0.035 | 0.319 | 0.966 | 0.517 | 1.803 |  |
|  | 100,000 to 499,999 | 0.074 | 0.159 | 1.077 | 0.789 | 1.470 | -0.688 | 0.238 | 0.503** | 0.315 | 0.802 |  |
| *Pseudo R-squared*^d^ | | *0.420**** | | | | | | | | | |  |
| *Note.* Weighted data. Ref: Reference category.  ^a^Final analysis sample with observed values for dependent variable.  ^b^The results differed between the original and full imputed datasets. In the original data, having no migration background was significantly associated with increased odds of being pre-frail (OR=1.415, [95% CI: 1.011, 1.981]).  ^c^The results differed between the original and full imputed datasets. In the original data, living in communities with 50,000 to 99,999 inhabitants was significantly associated with increased odds of being pre-frail (OR=1.776, [95% CI: 1.086, 2.903]).  ^d^Calculated as the mean value of the pseudo R-squared from all imputed datasets.  **p*<0.05; ***p*<0.01; ****p*<0.001. | | | | | | | | | | | | |

Table S3. Multinomial regression model of the associations between home environment and frailty level (*N* = 1,575^a^)

| **Variable** | | **Pre-frail relative to robust** | | | | | **Frail relative to robust** | | | | |  |
| --- | --- | --- | --- | --- | --- | --- | --- | --- | --- | --- | --- | --- |
|  |  | *Regression coefficient* | *Standard error* | *Odds ratio* | *95% CI* | | *Regression coefficient* | *Standard error* | *Odds ratio* | *95% CI* | |  |
| Age^b^ | | 0.047 | 0.022 | 1.048* | 1.003 | 1.096 | 0.054 | 0.031 | 1.055 | 0.994 | 1.121 |  |
| Sex (Ref: female) | | -0.021 | 0.167 | 0.979 | 0.706 | 1.358 | -0.103 | 0.248 | 0.902 | 0.554 | 1.468 |  |
| Socioeconomic Status | | 0.004 | 0.004 | 1.004 | 0.996 | 1.011 | 0.008 | 0.005 | 1.008 | 0.997 | 1.019 |  |
| Relationship status (Ref: in partnership) | | 0.151 | 0.165 | 1.163 | 0.842 | 1.608 | 0.048 | 0.245 | 1.050 | 0.649 | 1.698 |  |
| Migration background (Ref: no) | | 0.257 | 0.172 | 1.293 | 0.924 | 1.810 | -0.148 | 0.259 | 0.862 | 0.519 | 1.433 |  |
| Self-rated health status | | -0.612 | 0.115 | 0.542*** | 0.433 | 0.679 | -1.106 | 0.158 | 0.331*** | 0.243 | 0.451 |  |
| Number of chronic diseases | | 0.149 | 0.040 | 1.161*** | 1.073 | 1.257 | 0.214 | 0.053 | 1.238*** | 1.116 | 1.374 |  |
| Instrumental activities of daily living | | -2.278 | 0.343 | 0.102*** | 0.052 | 0.201 | -3.977 | 0.369 | 0.019*** | 0.009 | 0.039 |  |
| Walkability (Ref: rather high/high) | | -0.017 | 0.153 | 0.983 | 0.728 | 1.327 | -0.157 | 0.220 | 0.855 | 0.556 | 1.314 |  |
| Residential area | | -0.196 | 0.150 | 0.822 | 0.613 | 1.102 | -0.452 | 0.207 | 0.636* | 0.424 | 0.954 |  |
| Condition of interior living space | | -0.149 | 0.153 | 0.861 | 0.638 | 1.163 | -0.334 | 0.214 | 0.716 | 0.471 | 1.089 |  |
| Attachment to outdoor place  (Ref: rather close/very close) | | 0.517 | 0.188 | 1.677** | 1.160 | 2.426 | 0.931 | 0.255 | 2.536*** | 1.539 | 4.179 |  |
| Community type (Ref: ≥500,000) | |  |  |  |  |  |  |  |  |  |  |  |
|  | 5,000 to 49,999 | -0.128 | 0.229 | 0.880 | 0.561 | 1.380 | -1.307 | 0.360 | 0.271*** | 0.134 | 0.548 |  |
|  | 50,000 to 99,999^c^ | 0.502 | 0.248 | 1.653* | 1.016 | 2.688 | 0.114 | 0.341 | 1.120 | 0.575 | 2.184 |  |
|  | 100,000 to 499,999 | -0.008 | 0.164 | 0.992 | 0.719 | 1.369 | -0.642 | 0.251 | 0.526* | 0.322 | 0.862 |  |
| *Pseudo R-squared*^d^ | | *0.408**** | | | | | | | | | |  |
| *Note.* Weighted data. Ref: Reference category.  ^a^Final analysis sample with observed values for dependent variable.  ^b^The results differed between the original and full imputed datasets. In the original data, age was not associated with pre-frail status.  ^c^The results differed between the original and full imputed datasets. In the original data, living in communities with 50,000 to 99,999 inhabitants was not associated with pre-frail status.  ^d^Calculated as the mean value of the pseudo R-squared from all imputed datasets.  **p*<0.05; ***p*<0.01; ****p*<0.001. | | | | | | | | | | | | |

Table S4. Multinomial regression model of the associations between home environment and frailty level among community-dwelling very old adults (*N* = 1,436^a^)
